# Acute Compression of the Anterior Interosseous Nerve After a Proximal Both-Bone Forearm Fracture: A Case Report

**DOI:** 10.7759/cureus.64084

**Published:** 2024-07-08

**Authors:** Ophelie Lavoie-Gagne, Krystle R Tuano, Abhiram R Bhashyam

**Affiliations:** 1 Orthopaedic Surgery, Massachusetts General Hospital, Boston, USA; 2 Plastic Surgery, Massachusetts General Hospital, Boston, USA

**Keywords:** acute nerve palsy, hand and wrist surgery, orthopaedic traumatology, anterior interosseous nerve palsy, anterior interosseous nerve, case report, acute neurolysis, forearm fracture, nerve compression

## Abstract

Anterior interosseous nerve (AIN) syndrome is a rare condition characterized by isolated weakness in the flexor pollicis longus (FPL) muscle, sometimes accompanied by weakness in the index flexor digitorum profundus (FDP) muscle. In this clinical case report, an 18-year-old male presented with a right proximal both-bone forearm fracture that was sustained while playing soccer, with subsequent development of AIN palsy, without sensory deficits or progressive pain. Preoperative imaging was performed, showing a proximal third radius and mid-shaft ulna fracture. Given the progressive presentation of an acute AIN palsy, the patient was indicated for urgent operative intervention. During exploration and decompression of the AIN within the pronator tunnel, the nerve was found to be in continuity but was compressed by a large hematoma and the distal radial shaft. The patient recovered full median nerve function by his six-week postoperative examination and by his final follow-up recovered full range of motion with painless return to full activities.

In proximal or mid-shaft both-bone forearm fractures, a careful neurovascular exam is essential, as uncommon conditions like anterior interosseous syndrome (AIS) can present without obvious sensory deficits or pain. Potential etiology for traumatic AIN compression includes significant fracture displacement, soft tissue injury, active extravasation on advanced imaging, and/or clinical concern for compressive hematoma. Patients presenting with FPL and/or index FDP weakness in the absence of sensory deficits or pain on passive stretch may benefit from dedicated surgical exploration and decompression of the AIN to prevent irreversible nerve damage.

## Introduction

Anterior interosseous nerve (AIN) compression or injury is relatively uncommon. Kiloh and Nevin described two cases of spontaneous idiopathic interstitial AIN neuritis that self-resolved over the course of a year [1]. Since this early description in 1952, AIN neuritis has been reported in cases of compression due to hematoma [2], fluid extravasation after arthroscopy [3], constriction by the pronator teres [4], crutches [5], supracondylar humerus fractures in children [6], aneurysm following penetrating forearm trauma [7], anomalous pronator head anatomy [8], and constriction by fibrous bands of the flexor digitorum superficialis or vascular malformations [9]. While some patients may report pain at the pronator region of the forearm, the most consistent physical exam finding is weakness or paralysis of the flexor pollicis longus (FPL), with occasional involvement of the flexor digitorum profundus (FDP) to the index and/or long finger [3,9].

Atraumatic anterior interosseous syndrome (AIS) is most often managed with observation [9]. After eight weeks of observation without improvement and persistent palsy, some authors advocate for open neurolysis to decompress the AIN in the pronator interval [9-13]. In contrast, acute progressive nerve compression after trauma may benefit from expeditious decompression [2], similar to how carpal tunnel release is used to treat acute carpal tunnel syndrome [14]. In this case report, we describe the presentation, evaluation, and treatment of a patient who developed acute-onset progressive AIN palsy after a proximal both-bone forearm fracture, highlighting how careful serial examination and urgent surgical treatment can lead to successful neurolysis, fracture stabilization, and recovery of normal forearm and nerve function.

## Case presentation

**Figure 1:**
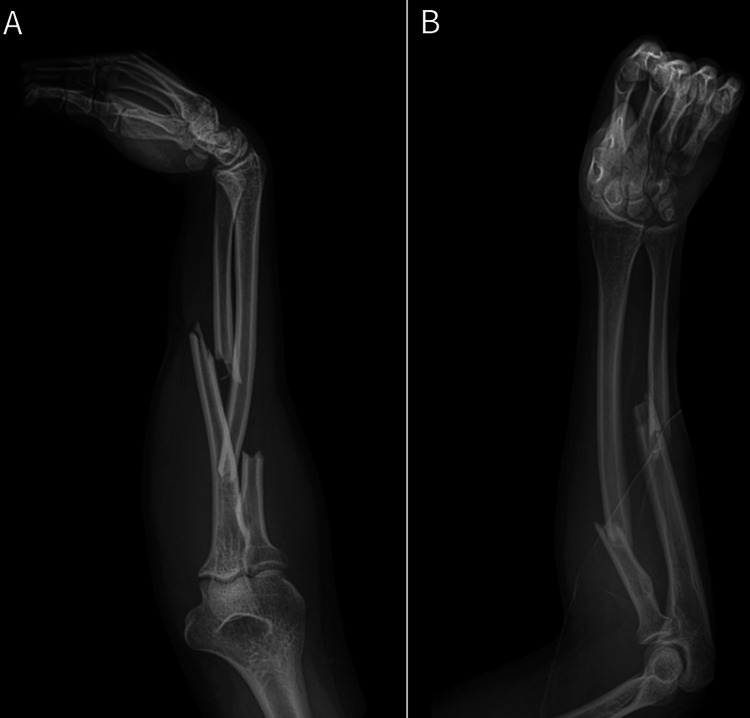
Pre-operative radiographs A) Lateral forearm radiograph; B) anteroposterior forearm radiograph

**Figure 2:**
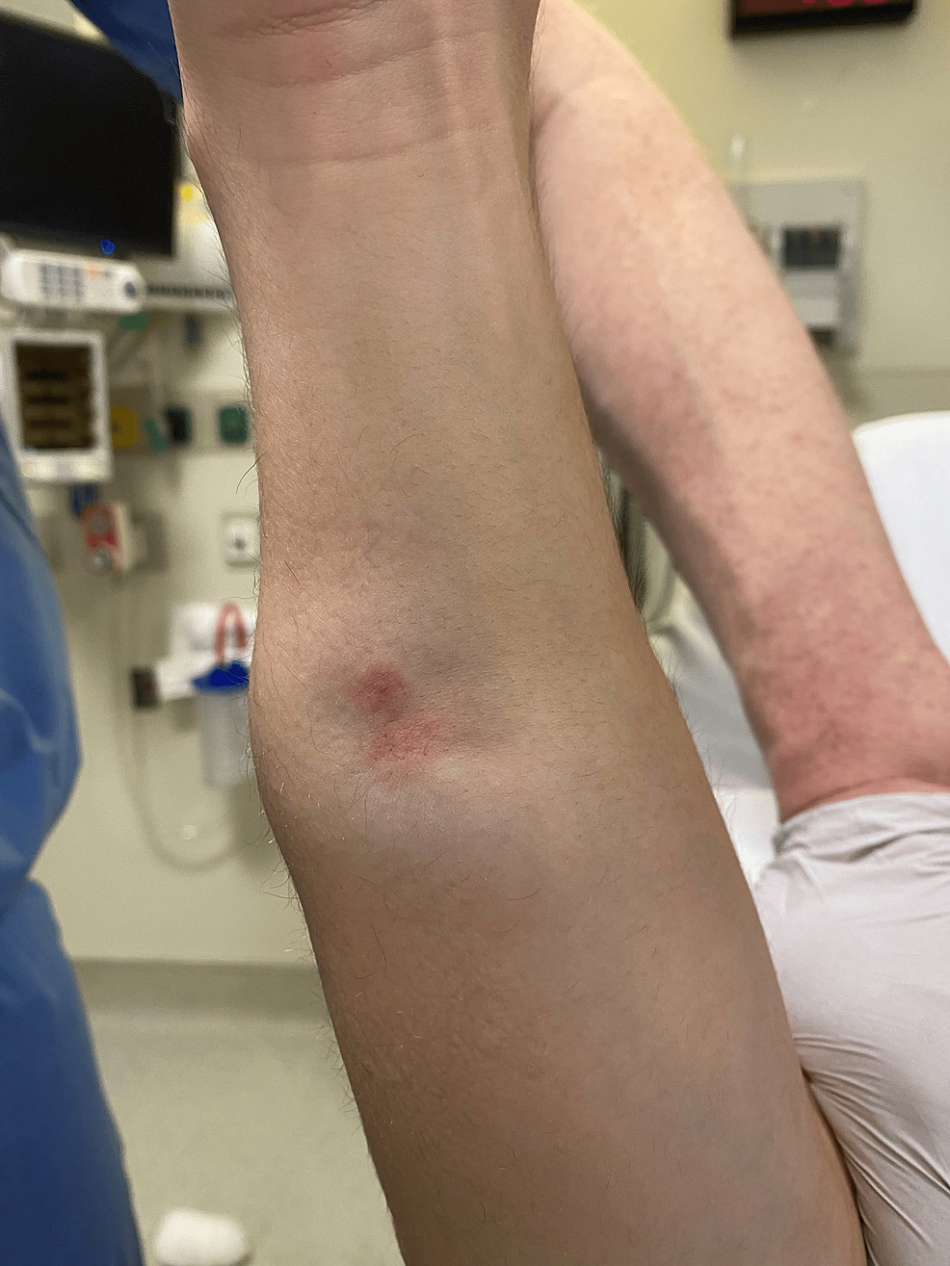
Preoperative clinical photo of skin dimpling

An 18-year-old, right-hand dominant, healthy male presented to the Emergency Department with a right proximal third both-bone forearm fracture sustained after a fall while playing soccer (Figure 1). His past medical history was notable for an ipsilateral distal radius fracture treated nonoperatively as a child, resulting in an apex dorsal distal radius malunion and associated apex volar plastic deformity of the ulna, without distal radio-ulnar joint (DRUJ) malreduction or instability. On initial presentation, he denied any numbness or paresthesias. His skin was intact, but he did have dimpling on the ulnar aspect of his forearm from ulna fracture displacement (Figure 2). Although strength was limited by pain, motor function was noted to be intact as he was able to activate the FPL, FDP to the index and long finger, extensor pollicis longus (EPL), and the first dorsal interosseous (DIO) muscle. He was placed into a plaster splint in situ, and the extremity was gently elevated.

The patient was admitted and had serial neurovascular examinations performed while awaiting operative intervention. He was re-examined four hours after the presentation, at which time his neurovascular exam was unchanged. Four hours later (eight hours after the initial presentation), he was re-examined by the senior surgeon (ARB) and was unable to fire FPL or FDP to his index and long fingers. Light touch sensibility in the median distribution remained unchanged. His pain remained responsive to oral analgesics, and he denied paresthesias. Due to acute and progressive loss of motor function in the AIN distribution, he was expeditiously taken to the operative room for median nerve/AIN exploration, possible neurolysis and/or repair, possible carpal tunnel release, prophylactic forearm fasciotomies, and surgical fixation of his both-bone forearm fracture.

**Figure 3:**
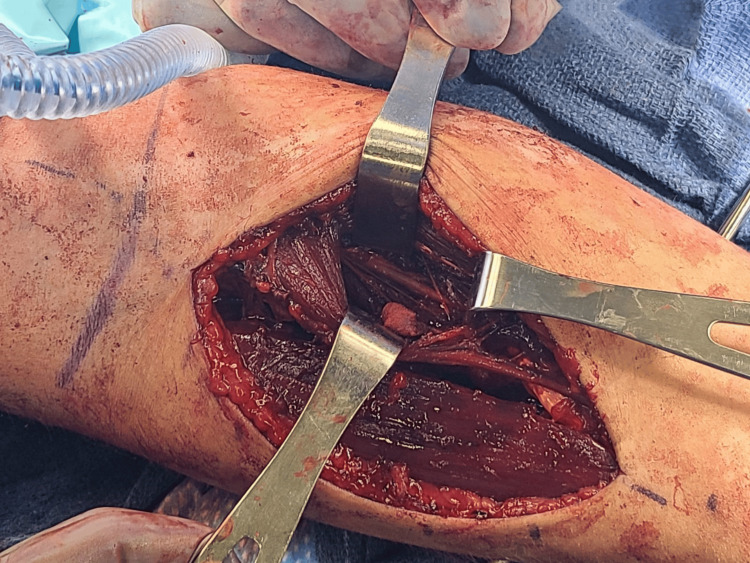
Pronator tunnel following decompression The pronator teres muscle was retracted radially by the retractor at the bottom of the photograph, while the flexor digitorum superficialis muscle belly was retracted ulnarly by the retractor at the top of the photograph, and the distal edge of the flexor superficialis tendinous arch was retracted distally by the retractor on the right of the photograph. The anterior interosseous nerve was visualized in continuity and fully decompressed in the pronator tunnel between these retractors.

After rapid sequence intubation, the right upper extremity was prepped and draped up to the axilla. Avoidance of paralysis was communicated with the anesthesiology team, given the anticipated use of intraoperative nerve stimulation to evaluate AIN function. A nonsterile tourniquet was inflated to 250 mmHg. Volar and dorsal fasciotomies were planned, with a proximal volar Henry approach and a dorsal incision over the mobile wad. Fasciotomy of the mobile wad was also completed during the approach. The two heads of the pronator teres muscle and the median nerve were identified. As the median nerve was traced distally, a hematoma was clearly found to be compressing the AIN branch within the pronator tunnel (Figure 3). The hematoma was evacuated, and the AIN was decompressed by releasing the pronator teres muscle fascia along its entire length. A nerve stimulator was then utilized to evaluate AIN function after decompression, on a 2 mA, 200 microsecond (ms) setting, which resulted in the firing of the FPL, FDP to the index finger, and FDP to the long finger.

The fracture was then fixated in the standard fashion. Dual plating was used for the radius, given its proximal location, and a single compression plate for the proximal ulna. Prophylactic McConnell fasciotomies of the volar and dorsal compartments were completed after fracture fixation.

Final fluoroscopic films demonstrated near-anatomic reduction, and the patient clinically had full pronation and supination following final fixation. The function of the median nerve was again confirmed to be intact with the use of a nerve stimulator on a 2 mA, 200 ms setting prior to closure. The patient was placed into a long-arm splint to neutralize the deforming force of the biceps tendon, given the proximal nature of the radial shaft fracture.

**Figure 4:**
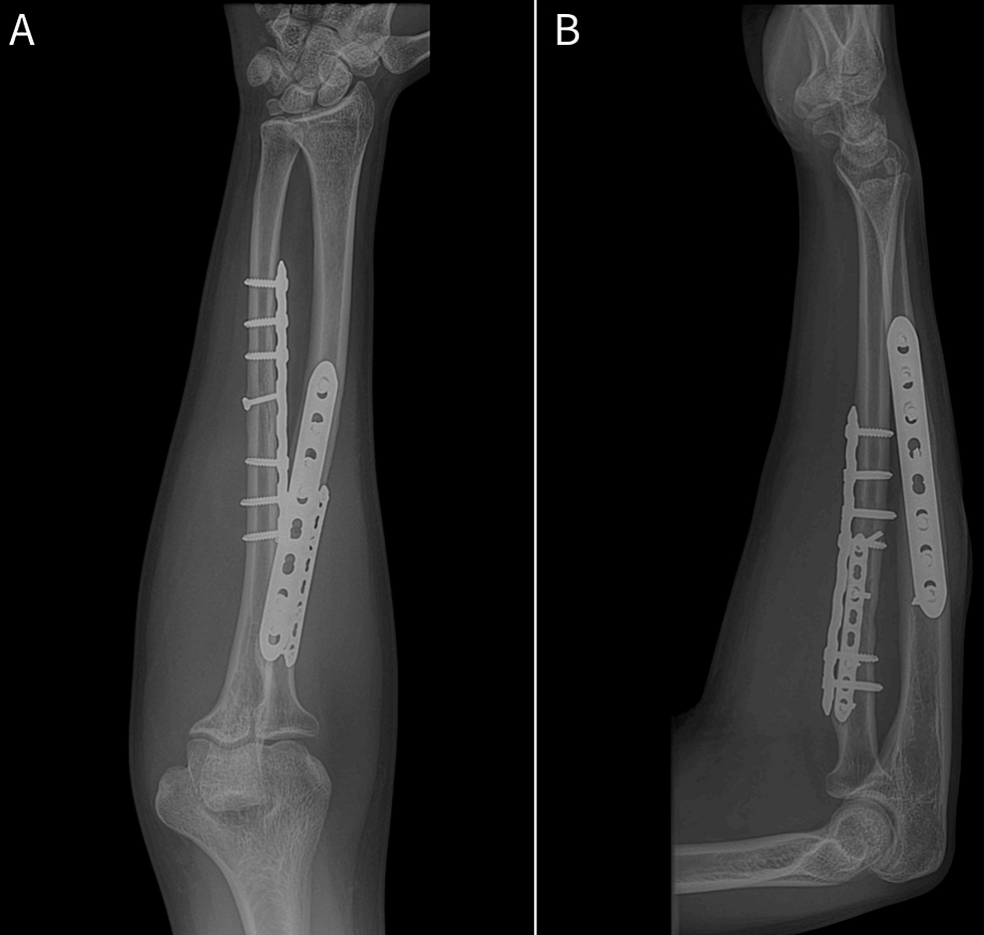
Radiographic union 12 weeks postoperatively A) Anteroposterior forearm radiograph; B) lateral forearm radiograph

**Figure 5:**
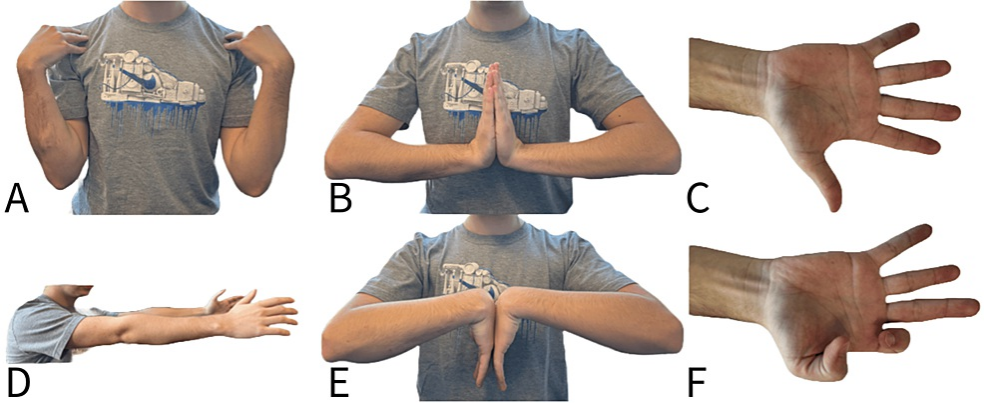
Full painless range of motion at final follow-up six months postoperatively A) Full elbow flexion; B) full wrist extension; C) full finger extension; D) full elbow extension; E) full wrist flexion; F) full flexion of flexor pollicus longus and flexor digitorum profundus to index demonstrating recovery of anterior interosseous nerve function

On postoperative day 1, the patient had recovered full strength of the index and long finger FDPs, but still had no active FPL function. His light-touch sensibility remained intact in the median, radial, and ulnar nerve distributions. Two weeks postoperatively, he recovered three out of five strength in his FPL, with maintained FDP strength. Six weeks postoperatively, he recovered full strength of his FPL and had regained full pronation-supination. At three months postoperatively, his fractures were well-healed clinically and radiographically. He had a full return to all activities without limitation (Figures 4-5).

## Discussion

This report describes a case of AIS in an 18-year-old male who sustained a right proximal third both-bone forearm fracture, treated with expeditious surgical decompression of a traumatic hematoma in the pronator tunnel compressing the AIN. The patient recovered full function and painless return to all activities by three months. Patients presenting with FPL and/or index FDP weakness in the absence of sensory deficits or pain on passive stretch may benefit from dedicated surgical exploration and decompression of the AIN to prevent irreversible nerve damage.

The differential diagnosis of neurological complications secondary to both-bone forearm fractures includes compartment syndrome, acute carpal tunnel syndrome, traumatic neuropraxia, and AIS/pronator syndrome. Compartment syndrome is the most critical diagnosis to identify acutely as it requires emergent operative intervention. Acute carpal tunnel syndrome and AIS in the setting of trauma can be a result of focal compression due to hematoma and/or mass effect from fractures or dislocations and similarly indicate the patient for urgent surgical intervention to prevent irreversible nerve injury. AIS can be differentiated from acute carpal tunnel syndrome by examining median nerve sensibility (intact in AIS) and differentiated from concomitant tendon rupture by evaluating tenodesis (intact in AIS) [15]. Traumatic neuropraxia may be due to a combination of the mechanism of injury and the fracture [6,16].

In this clinical case, the treatment team had the benefit of evaluating the patient within a few hours of injury, at which time the patient was neurovascularly intact. Over serial examinations, the patient remained comfortable with soft compartments but developed a progressively isolated motor deficit in the AIN distribution, with a clear constellation of symptoms consistent with AIS. Of note, most of the current literature on AIS focuses on subacute presentations, where patients note several months of weakness that may be accompanied by forearm pain in the setting of nerve compression. Electrophysiological studies are often used for diagnosis and may demonstrate abnormalities in the median nerve distribution distal to the elbow but proximal to the wrist [9,12,15,17]. The pathophysiological mechanism of symptoms in AIS is postulated to be due to neuritis, focal compression, or a combination of both [9,12,15,17]. In the absence of trauma, there remains controversy in management and the timing of surgical exploration if symptoms do not resolve spontaneously [10,11,17,18]. Patients who benefit from surgical intervention typically have focal sites of compression [7]. In the setting of trauma, hematoma can cause local compression, and if left untreated, lead to progressive intraneural ischemia with irreversible neural injury [19]. Prior case reports of acute AIS have reported similar resolution of motor deficits after surgical decompression [2,6,7,16]. Motor nerve branches to the FPL and index FDP arise from the proximal portion of the AIN, approximately 30 mm distal to the branch point of the AIN from the median nerve [18]. Intraoperative nerve stimulation can be a helpful adjunct to assessing nerve function and prognosis [20].

## Conclusions

In proximal or mid-shaft both-bone forearm fractures, a careful neurovascular exam is essential, as uncommon conditions like AIS can present without obvious sensory deficits or pain. Potential etiology for traumatic AIN compression includes significant fracture displacement, soft tissue injury, active extravasation on advanced imaging, and/or clinical concern for compressive hematoma. Patients presenting with FPL and/or index FDP weakness in the absence of sensory deficits or pain on passive stretch may benefit from dedicated surgical exploration and decompression.
